# Association of proton pump inhibitors with gastric and colorectal cancer risk: A systematic review and meta-analysis

**DOI:** 10.3389/fphar.2023.1129948

**Published:** 2023-03-16

**Authors:** Huizhu Guo, Ruiqiu Zhang, Pei Zhang, Zhaoyang Chen, Yuqing Hua, Xin Huang, Xiao Li

**Affiliations:** Department of Clinical Pharmacy, The First Affiliated Hospital of Shandong First Medical University and Shandong Provincial Qianfoshan Hospital, Shandong Engineering and Technology Research Center for Pediatric Drug Development, Shandong Medicine and Health Key Laboratory of Clinical Pharmacy, Jinan, China

**Keywords:** proton pump inhibitor, gastric cancer, colorectal cancer, systematic review, meta-analysis

## Abstract

**Background:** Proton pump inhibitors (PPI) are generally considered to be one of the well-established prescription drug classes and are commonly used to treat most acid-related diseases. However, a growing body of literature showing an association between gastric and colorectal cancer risk and PPI use continues to raise concerns about the safety of PPI use. Therefore, we aimed to investigate the association between proton pump inhibitor use and risk of gastric and colorectal cancer.

**Methods:** We collected relevant articles using PubMed, Embase, Web of Science and Cochrane library from 1 January 1990 to 21 March 2022. The pooled effect sizes were calculated based on the random-effects model. The study was registered with PROSPERO (CRD42022351332).

**Results:** A total of 24 studies (*n* = 8,066,349) were included in the final analysis in the screening articles. Compared with non-PPI users, PPI users had a significantly higher risk of gastric cancer (RR = 1.82, 95% CI: 1.46–2.29), but not colorectal cancer (RR = 1.22, 95% CI: 0.95–1.55). Subgroup analysis showed that there was a significant positive correlation between the use of PPI and the risk of non-cardiac cancer (RR = 2.75, 95% CI: 2.09–3.62). There was a significant trend between the duration dependent effect of PPI use and the risk of gastric cancer (<1 year RR = 1.56, 95% CI: 1.30–1.86; 1–3 years RR = 1.75, 95% CI: 1.28–2.37; >3 years RR = 2.32, 95% CI: 1.15–4.66), but not colorectal cancer (≤1 year RR = 1.00, 95% CI: 0.78–1.28; >1 year RR = 1.18, 95% CI: 0.91–1.54; ≥5 years RR = 1.06, 95% CI: 0.95–1.17).

**Conclusion:** We found that PPI use increased gastric cancer risk, but not colorectal cancer risk. This result may be biased due to confounding factors. More prospective studies are needed to further validate and support our findings.

**Systematic Review Registration**: [https://www.crd.york.ac.uk/prospero/display_record.php?ID=CRD42022351332], identifier [CRD42022351332].

## 1 Introduction

Gastric cancer (GC) is a common digestive system disease with more than 1 million new diagnoses and an estimated 780,000 deaths each year, accounting for 5.7% of global cancer incidence and 8.2% of deaths (Bray et al., 2018; Stoffel and Murphy, 2020). Despite global morbidity and mortality declines over the past 5 decades, gastric cancer is the fifth most commonly diagnosed cancer and the third leading cause of cancer-related death worldwide. *Helicobacter pylori* (H. pylori) infection is a major risk factor for gastric cancer, and almost 90% of new cases of non-cardiogenic gastric cancer are attributed to it (Yusefi et al., 2018; Machlowska et al., 2020). Colorectal cancer (CRC) is a common but heterogeneous disease with the third highest incidence rate (10.2%) and the second highest mortality rate (9.2%) worldwide (Stoffel and Murphy, 2020). The global burden of CRC is projected to increase by 60% by 2030, adding 2.2 million new cases and 1.1 million deaths (Arnold et al., 2017; Bray et al., 2018). Although the pathogenesis of colorectal cancer is not fully understood, there is compelling evidence that genetics, environmental exposures, and lifestyle factors are associated with an increased risk of colorectal cancer (Stoffel and Murphy, 2020; Baidoun et al., 2021).

In the past few decades, proton pump inhibitors have been widely used clinically for acid-related diseases (William and Danziger, 2016; Vaezi et al., 2017). Although PPI are generally considered to be effective and safe, they have many potential risks (Savarino et al., 2018; Elias and Targownik, 2019). PPI-induced hypergastrinemia has been identified as a possible risk factor for gastrointestinal cancers including gastric cancer (GC) and colorectal cancer (CRC) (Schenk et al., 1998; Laine et al., 2000; Watson et al., 2002). Serum gastrin levels are elevated, which may lead to proliferative changes in the gastric mucosa and growth of colonic epithelial cells over the long term (Eissele et al., 1997; Lundell et al., 2015). Several epidemiological studies have evaluated the association between long-term use of PPI and the risk of gastric and colorectal cancer. However, the association between PPI use and risk of GC and CRC remains controversial, with conflicting findings in the literature. Some epidemiological studies have found an increased risk of gastric and colorectal cancer in proton pump inhibitor users (Wennerström et al., 2017; Niikura et al., 2018; Brusselaers et al., 2019), while other studies have found no evidence of a significantly increased risk (van Soest et al., 2008; Poulsen et al., 2009; Lee et al., 2020). Therefore, we performed a systematic review and meta-analysis of existing retrospective cohort studies and case-control studies to objectively assess the potential risk of proton pump inhibitors for gastric and colorectal cancer development.

## 2 Materials and methods

This systematic review and meta-analysis was conducted according to the Preferred Reporting Items for Systematic Reviews and Meta-analyses (PRISMA) reporting guideline (Moher et al., 2009). The study protocol for this systematic review was registered in the PROSPERO international prospective register of systematic reviews (Registration number: CRD42022351332).

### 2.1 Data sources and search strategy

We selected relevant studies published between 1 January 1990 and 21 March 2022 to assess the association between PPI and GC and CRC risk by searching PubMed, Embase, Cochrane Library, Web of Science. The search strategy appears in Supplementary Figure S1. Manual searches of references were conducted to identify other reports from a list of review articles and original research.

### 2.2 Study selection

Studies were included if they met the following criteria: 1) case-control or cohort study; 2) the study compared at least two independent groups (i.e., PPI use and PPI non-use groups); 3) clear results with gastric or colorectal cancer; 4) literature that can directly or indirectly provide raw data to calculate parameters such as risk ratio (RR), risk ratio (HR) or odds ratio (OR), and 95% confidence interval (CI); 5) studies written in English. Articles that meet one of the following criteria are excluded: 1) duplicate publications; 2) studies for which data of interest cannot be retrieved or calculated; 3) non-clinical trials (animal experiments, etc.); 4) systematic review articles, meta-analyses, editorials, protocols and case reports. Two independent investigators reviewed study titles and abstracts and searched for studies that met inclusion criteria for a comprehensive assessment. Any disagreements at this stage were resolved by discussion and consensus with the third reviewer.

### 2.3 Data extraction

Data extraction was performed independently by two investigators, results were compared, and discussions were conducted to resolve disagreements. The following information was extracted: first author name, year of publication, region, study design, observation period, percentage of sex, size and mean age of the included population, lag time, primary and secondary outcomes. Two investigators independently assessed the methodological quality of included studies using the Newcastle-Ottawa Scale. Three areas of research were evaluated, including selection of participants, comparability of study groups, and identification of outcomes of interest, to assign star ratings representing the quality of the study (Oremus et al., 2012). The overall quality score is nine points. We consider a study to be of high quality if it has a score of ≥7 (Supplementary Table S1).

### 2.4 Statistical analyses

To calculate the pooled RR with 95% CI, we used the adjusted RR and 95% CI reported in each article where possible. Study weights were calculated using the inverse variance method. Due to the low incidence (<10%) of gastric and colorectal cancers, RR, OR, and HR are considered equivalent measures for risk assessment (Grant, 2014). Data analysis was performed on adjusted ratios whenever available, and we used unadjusted ratios if only unadjusted data were available. The Cochrane chi-square test was used to judge the heterogeneity of the included studies according to the *p*-value and I^2^ value. Heterogeneity among studies was classified as low (I^2^: <25%), moderate (I^2^: 50%–75%), and high (I^2^: >75%), respectively (Higgins et al., 2003). We considered I^2^>50% and *p* < 0.05 to represent significant heterogeneity, and selected a random-effects model (DerSimonian-Laird method) for analysis (Singh et al., 2017). In sensitivity analyses, the effect of individual studies on the pooled estimates was assessed by excluding included studies one by one. Furthermore, we performed a subgroup analysis to further explore potential sources of heterogeneity, subgroup analysis of GC including study design (case-control study, nested case-control study, cohort study), events (<1,000, ≥1,000), population (≤ 10,000, >10,000, >50,000), mean age (<65, ≥65), region (North America, Europe, Asia), Newcastle-Ottawa Scale (NOS) score (<7, ≥7), lag time (<1 year, ≥1 year), year of publication (<2010, ≥2010), duration of PPI use (<1 year, 1–3 years, >3 years), GC site (adenocarcinoma, cardia, non-cardia), eradication of *H. pylori* infection, subgroup analysis of CRC including study design (case-control study, nested case-control study, cohort study), events (<1,000, ≥1,000), population (≤ 10,000, >10,000, >50,000), mean age (<65, ≥65), region (North America, Europe, Asia), Newcastle-Ottawa Scale (NOS) score (< 7, ≥7), lag time (<1 year, ≥1 year), year of publication (<2010, ≥2010), duration of PPI use (<1 year, ≥1 year, ≥5 years), adjusted for CRC risk (use of NSAIDs/aspirin, BMI, follow-up duration). We assessed publication bias using Begg’s test and Egger’s test. All statistical analyses were performed using Stata 14.0 (Stata Inc, University of Texas Station, United States).

## 3 Results

### 3.1 Study selection

**FIGURE 1:**
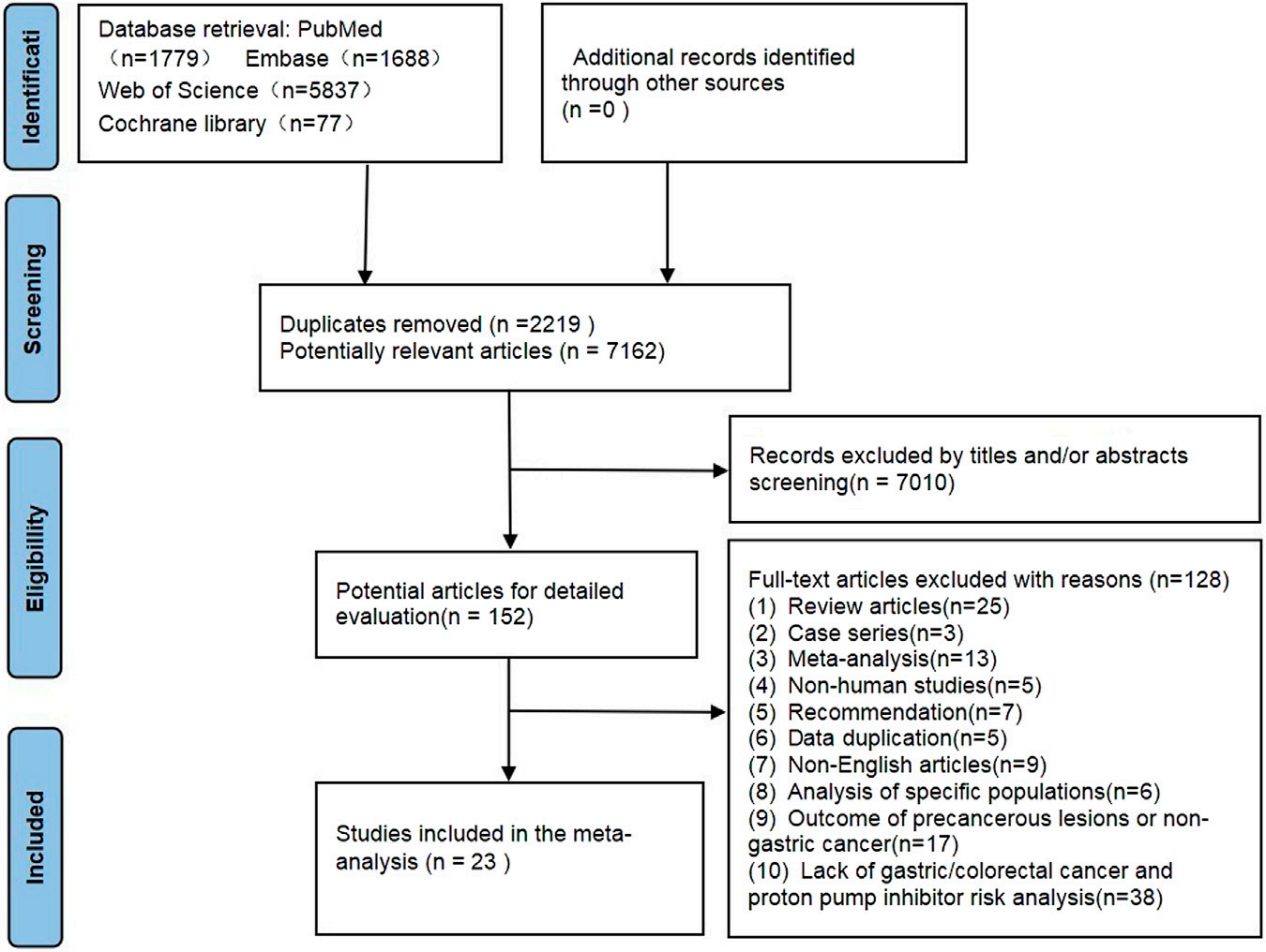
Flow chart of study selection process.

We retrieved 9,381 potentially relevant records through data-base searches, of which 2,219 duplicate records were removed. 152 records were retained for full-text review after reviewing the title and abstract (Figure 1.). Eventually, 22 eligible articles were included in our analysis (Garcia Rodriguez et al., 2006; Robertson et al., 2007; Yang et al., 2007; Tamim et al., 2008; van Soest et al., 2008; Chubak et al., 2009; Poulsen et al., 2009; Lai et al., 2013; Hwang et al., 2017; Wennerström et al., 2017; Cheung et al., 2018; Niikura et al., 2018; Brusselaers et al., 2019; Babic et al., 2020; Kuiper et al., 2020; Liu et al., 2020; Seo et al., 2021; Abrahami et al., 2022a; Abrahami et al., 2022b), Lai (Lai et al., 2013) and Lee (Lee et al., 2020) studies included two independent studies. Therefore, we analyzed the association between PPI use and gastric cancer, colorectal cancer risk in 24 studies, including 13 studies on gastric cancer and 11 on colorectal cancer.

### 3.2 Study characteristics

Table 1 and table 2 summarized the characteristics of the included studies. The 24 studies had a total of 8,066,349 participants. Countries and regions of origin include the United States (Chubak et al., 2009; Babic et al., 2020; Lee et al., 2020), Denmark (Robertson et al., 2007; Poulsen et al., 2009; Wennerström et al., 2017), the United Kingdom (Garcia Rodriguez et al., 2006; Yang et al., 2007; Liu et al., 2020; Abrahami et al., 2022a; Abrahami et al., 2022b), Netherlands (van Soest et al., 2008; Kuiper et al., 2020), Canada (Tamim et al., 2008), Taiwan (Lai et al., 2013; Brusselaers et al., 2019; Lai et al., 2019; Lei et al., 2021), Hong Kong (Cheung et al., 2018), Japan (Niikura et al., 2018), and South Korea (van Soest et al., 2008; Seo et al., 2021). All studies in the analysis were adjusted for different potential confounders. *Helicobacter pylori* infection status is an important potential confounder for gastric cancer, and three studies were considered to eradicate *H. pylori* (Wennerström et al., 2017; Niikura et al., 2018; Brusselaers et al., 2019). Supplementary appendix two shows the Newcastle-Ottawa Scale quality assessment of the included studies. We considered a NOS score ≥7 to be high-quality studies, with three studies rated as moderate-quality studies (scores 5–6). The mean of eligible studies was 7.3, thus indicating a high quality of included studies.

**TABLE 1:** Characteristics of the included studies on the use of PPI and risks of Gastric Cancer.

First author, year	Area	Study design	Period of recruitment	Sex (female, %)	Range of age	PPI (n)	Non-PPI (n)	Non-GC (n)	GC (n)	Lag time	NOS score	Adjustment for covariates
Garcia Rodriguez 2006	The United Kingdom	Case– control	1994–2001	N/A	N/A	442	9,851	9,786	507	1 year	8	age, sex, calendar year, smoking, alcohol, BMI, UGI disorder, hiatal hernia, GU, DU, dyspepsia
Tamim 2008	Canada	Nested case- control	1995–2003	47.9	N/A	1,299	6,930	7,158	1,071	6 months	8	number of prescriptions to any drug, totallength of hospitalizations and number of visits to GPs, specialists and emergency rooms
Poulsen 2009	Denmark	Cohort	1990–2003	53	40–84	18,790	262,082	280,329	543	1 year	7	age, gender, calendar period, gastroscopy (**≥**1 year before censoring events), use of NSAIDs and *H. pylori* eradication
Cheung 2017	Hong Kong	Cohort	2003–2012	53.5	**≥**18	3,271	60,126	63,244	153	6 months	8	Age, sex, comorbidities, smoking, alcohol, aspirin/NSAIDs, H2RA, *H. pylori*
Niikura 2017	Japan	Cohort	1998–2017	44	N/A	118	415	512	21	N/A	6	age, sex, comorbidities, aspirin/NSAIDs, *H. pylori*
Wennerström 2017	Denmark	Cohort	1996–2017	55.5	all ages	1,179,202	384,658	1,561,810	2050	1 year	7	Age, sex, municipality
Lai 2018	Taiwan	Case-control	2000–2013	34.4	20–84	539	759	649	649	1 year	6	Age, sex, comorbidities
Brusselaers 2019	Sweden	Cohort	2005–2012	58.5	**≥**18	796,492	20,210	814,761	1941	1 year	7	age, sex, calendar period
Liu (PCCIU) 2020	The United Kingdom	Case-control	1999–2011	42.9	all ages	1,542	6,513	6,607	1,448	1–2 years	7	age, sex, deprivation, BMI, alcohol, smoking, comorbidities at baseline, statins/aspirin use at baseline
Liu (United Kingdom Biobank) 2020	The United Kingdom	Cohort	2006–2010	54	40–70	46,146	425,633	471,529	250	1–2 years	7	age, sex, deprivation, BMI, alcohol, smoking, comorbidities at baseline, statins/aspirin use at baseline
Lee 2020	Northern California	Nested case-control	1995–2011	25.3	**≥**18	937	10,839	10,543	1,233	2 years	8	age, sex, race, chronic alcohol consumption, smoking, BMI, family history of gastric cancer, *H. pylori* infection, gastric intestinal metaplasia and dysplasia, atrophic gastritis, Barrett’s esophagus, and peptic ulcer disease
Seo 2021	Korea	Cohort	2002–2013	52.1	N/A	11,741	11,741	23,324	158	2 years	8	age, sex, smoking history, alcohol consumption, comorbidities, previous medications including statins, metformin, aspirin, NSAIDs, clopidogrel and H2RA before the index date

**TABLE 2:** Characteristics of the included studies on the use of PPI and risks of Colorectal Cancer.

First author, year	Area	Period of recruitment	Study design	Male	Age (≥ years)	PPI (n)	Non-PPI (n)	CRC (n)	Non-crc (n)	Lag time	Study quality (NOS)	Adjustment for covariates
Yang 2007	The United Kingdom	1987–2002	Nested case-control	Cases: 54.5% Controls: 44.2%	50	5,902	42,822	4,432	44,292	1 year	9	age, sex, smoking, BMI, H2RA use, HRT use, NSAID/aspirin use, and colonoscopy or flexible sigmoidoscopy
Robertson 2007	Denmark	1989–2005	Nested case-control	50.20%	71 (mean)	2,987	58,492	5,589	55,890	1 year	8	age, sex, place of residence aspirin/NSAID use, H2RA use, antidiabetic use, statin use, history of cholecystectomy, and alcoholism
van Soest 2008	Netherlands	1996–2005	Case-control	Cases: 50.7% Controls: 51.8%	Cases: 69.5 Controls: 69.3 (mean)	778	7,606	594	7,790	1 year	9	age, sex, calendar time, follow-up duration, comorbidity
Chubak 2009	The United States of America	2000–2003	Case-control	48.40%	**≥**40	25	953	498	480	1 year	7	age, sex, follow-up duration, calendar time
lai 2013	Taiwan	2000–2010	Nested case-control	Cases: 57.2% Controls: 57.2%	**≥**20	NA	NA	3,989	15,956	N/A	6	age, sex, alcoholism, diabetes mellitus, colorectal adenomas, IBD, H2RA use, statin use, aspirin/NSAID use
Hwang 2017	Korea	2007–2013	Cohort	PPI users: 54.3% Non-users: 53.4%	**≥**40	49,520	401,764	5,304	445,980	1 year	8	age, sex, smoking, alcohol, BMI, consumption, physical activity, type 2 diabetes, CCI score, aspirin use, metformin use, stain use, socioeconomic statu
Lei 2020	Taiwan	1999–2011	Cohort	PPI users: 52.5% Non-users: 52.2%	**≥**20	45,382	45,382	265	90,499	1 year	9	age, sex, comorbidities, and medication
Babic 2020	The United States of America	1988–2015	Cohort	PPI users: 29.1% Non-users:< 12.8%	25–75	162,654	13,205	1,255	174,604	1 year	6	age, physical activity, BMI, family history of CRC, alcohol, smoking, history of lower endoscopy, caloric intake, vitamin D, calcium intake, regular aspirin use, folate intake, menopausal hormone therapy use, and red meat as main dish
Kuiper 2020	Netherlands	2007–2014	Case-control	PPI users: 58% Non-users: 58%	**≥**40	5,202	4,688	7,912	1978	6 years	7	age, sex, use of antidiabetics, aspirins, H2 receptor antagonist, NSAIDs, statins
Lee 2020	The United States of America	1996–2016	Nested case-control	Cases: 51.2% Controls: 51.9%	**≥**18	12,219	166,498	18,595	160,122	2 years	8	age, sex, race/ethnicity, medical facility, enrollment duration, chronic alcohol consumption, smoking, BMI, family history of colorectal cancer, Crohn’s disease, ulcerative colitis, and colonoscopy utilization
Abrahami 2021	The United Kingdom	1990–2018	Cohort	Cases 52.6% Controls: 52.6%	**≥**18	1,293,749	1,294,713	2,580,439	8,023	1 year	8	Age, sex, alcoholrelated disorders, smoking status, BMI, type 2 diabetes, hypertension, chronic obstructive pulmonary disease, cancer (other than non-melanoma skin cancer), Crohn’s disease, UC, other IBD, GI polyps, cholecystectomy and solid organ transplant. Acid suppressant drug use drugs previously associated with colorectal cancer incidence

### 3.3 Association of PPI use with gastric cancer risk

**FIGURE 2:**
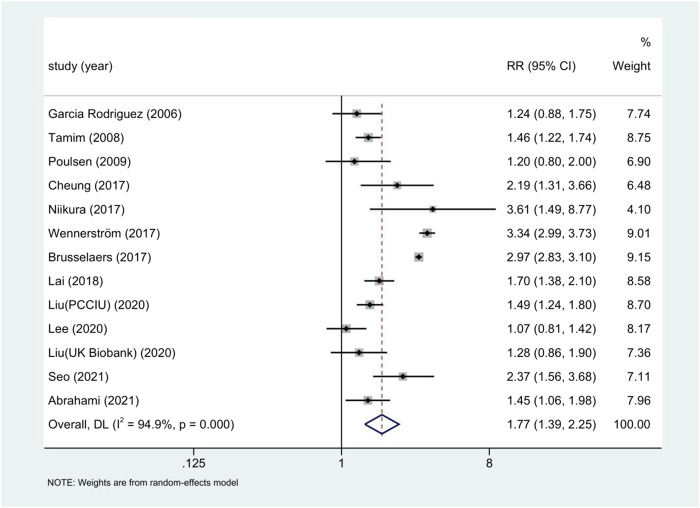
Forest plot of long-term use of PPI and the risk of Gastric Cancer.

The relationship between long-term PPI use and gastric cancer risk is shown in Figure 2. Thirteen studies of 4,431,863 participants showed that long-term PPI use was significantly associated with an increased risk of gastric cancer, with an overall RR of 1.82 (95% CI: 1.46–2.29), with high heterogeneity (*p* < 0.05; I^2^ = 94.9%).

**FIGURE 3:**
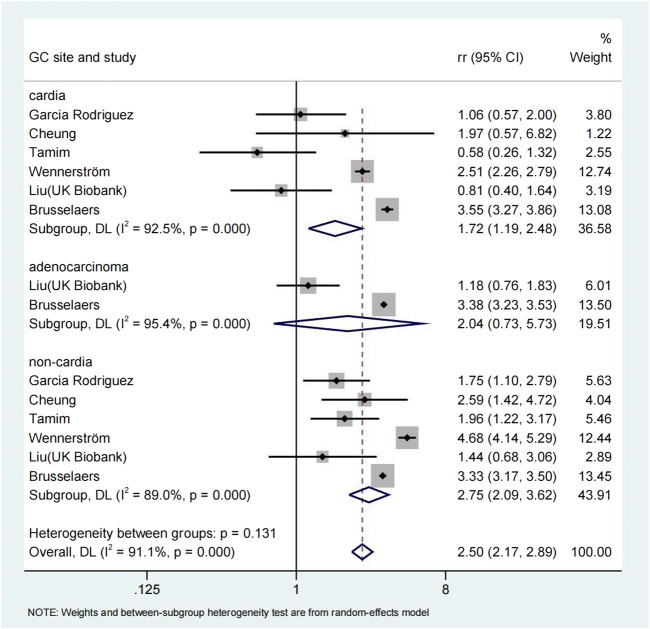
Association between the use of PPI and risks of Gastric Cancer stratified by Gastric Cancer site.

**FIGURE 4:**
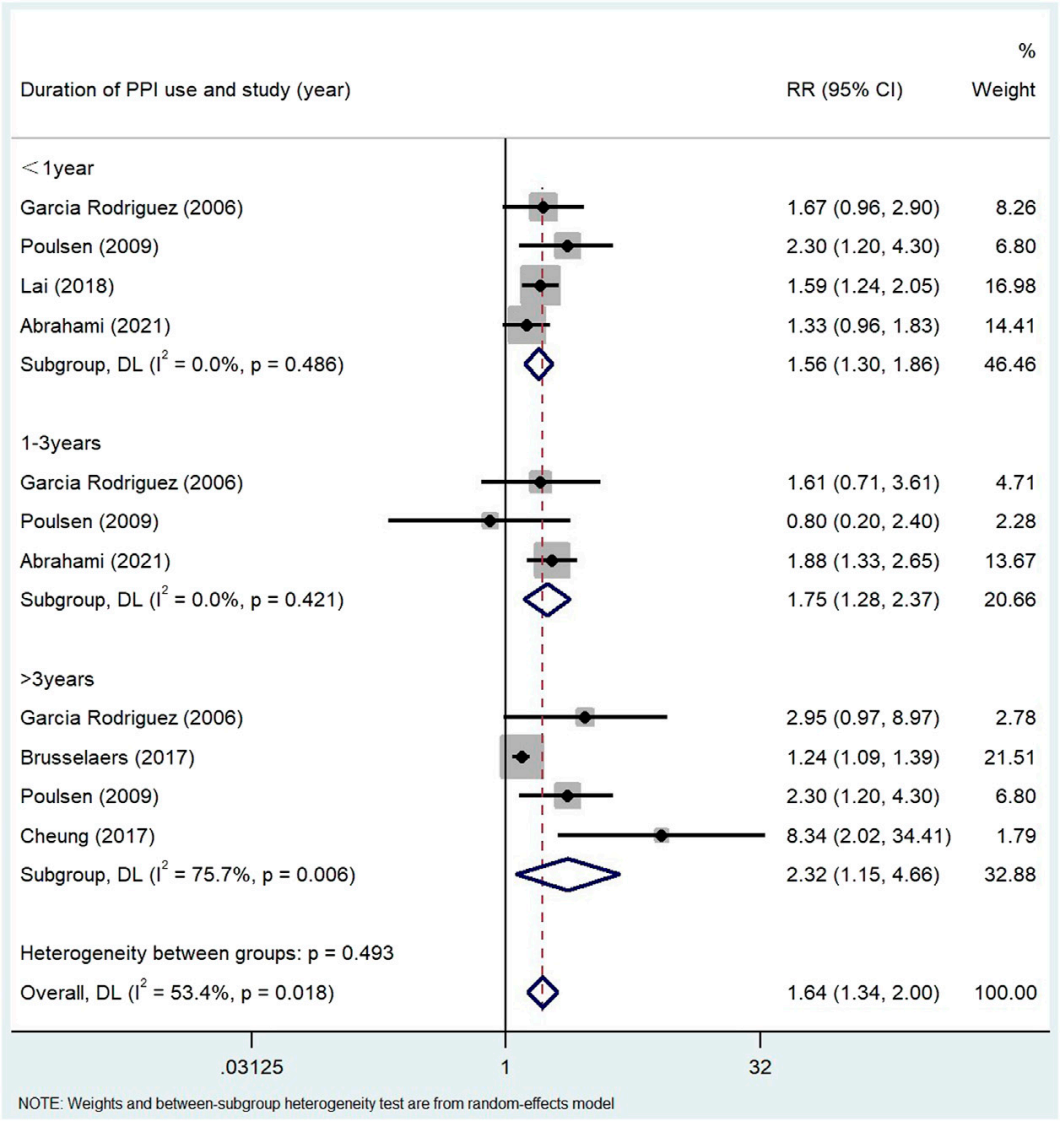
Association between the use of PPI and risks of Gastric Cancer stratified by duration of PPI use.

**FIGURE 5:**
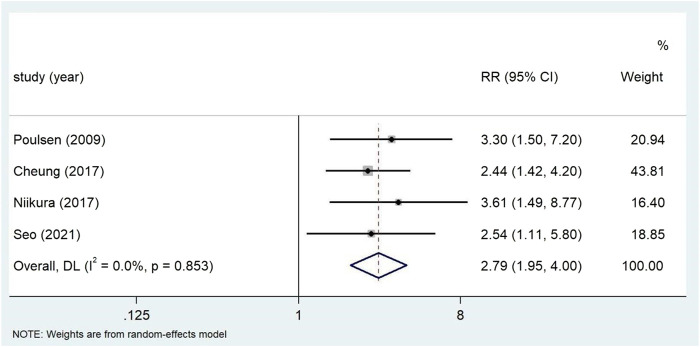
PPI users after *Helicobacter pylori* eradication.

The use of PPI was always associated with the risk of gastric cancer in subgroup analysis (Table 3). The risk of gastric cancer in nested case-control study using PPI was higher than that in cohort study (RR = 2.15, 95% CI: 1.71–2.70; RR = 1.27, 95% CI: 0.94–1.72). We determined the GC site by positioning according to the International Classification of Diseases (ICD) code. Stratified analysis based on the GC site showed that there was a significant positive correlation between the use of PPI and the risk of non-cardia cancer (RR = 2.75, 95% CI: 2.09–3.62) compared with cardia and adenocarcinoma (RR = 1.72, 95% CI: 1.19–2.48; RR = 2.04, 95% CI: 0.73–5.73) (Figure 3). Hierarchical analysis based on the duration of PPI showed that there was a significant trend between the duration dependent effect of PPI use and the risk of gastric cancer (<1 year RR = 1.56, 95% CI: 1.30–1.86; 1–3 years RR = 1.75, 95% CI: 1.28–2.37; >3 years RR = 2.32, 95% CI: 1.15–4.66) (Figure 4). PPI use was still positively associated with gastric cancer risk after eradication of potential confounders of *H. pylori* infection (RR = 3.09, 95% CI: 2.74–3.49) (Figure 5). Subgroup analysis shows that the heterogeneity can be partly explained by the study design (RR = 1.52, 95% CI: 1.19–2.48, I^2^< 50%), region (RR = 2.04, 95% CI: 1.58–2.63, I^2^< 50%), population (RR = 1.58, 95% CI: 1.36–1.83, I^2^< 50%) and mean age (RR = 1.56, 95% CI: 1.34–1.81, I^2^< 50%)and all were related to the risk of gastric cancer. However, the results of subgroup analysis are still highly heterogeneous due to the influence of potential confounding factors. Sensitivity analysis shows that the study of Wennerström (Wennerström et al., 2017) has the greatest impact on the overall results (Supplementary Figure S1). After rejecting Wennerström’s study, the RR was 1.50 (95% CI: 1.31–1.71, I^2^ = 49.8%). The results still showed that long-term use of PPI was associated with the risk of gastric cancer. Egger’s test and Begg’s test (Egger et al., 1997) showed no publication bias in the study (*p*_Egger’s test_ = 0.209, *p*_Begg’s test_ = 0.115).

**TABLE 3:** Subgroup analysis of Proton Pump Inhibitor Use and the Risk of Gastric Cancer.

Stratified by study design	NO.of studies	PPI users (n)	Non-users (n)	Relative Risk (95% CI)	I^2^ (%)	*p*-Value	Heterogenerty, *p*-value
case-control	3	2,523	17,123	1.52 (1.31.1.76)	19.3	0.289	0.01
Nested case-control	2	2,236	17,769	1.27 (0.94.1.72)	70.3	0.067	
Cohort	8	3,029,041	1,363,171	2.15 (1.71.2.70)	88.1	0.000	
**Events**							
**<**1,000	6	69,306	758,866	1.55 (1.23.1.95)	48.4	0.085	0.319
**≥**1,000	7	2,964,494	639,197	1.88 (1.39.2.56)	96.5	0.000	
**Population**							
**>**10,000	2	1,379	20,690	1.14 (0.91.1.41)	0	0.515	0.001
**≤**10,000	4	3,498	14,617	1.58 (1.36.1.83)	38.1	0.183	
**>**50,000	7	3,028,923	1,362,756	2.09 (1.65.2.64)	89.8	0.000	
**Mean age**							
**<**65	4	1,007,083	532,255	1.70 (1.25.2.50)	53.2	0.093	0.608
**≥**65	2	1838	7,689	1.56 (1.34.1.81)	15.1	0.278	
**Region**							
Europe	8	2,220,702	1,293,973	1.72 (1.28.2.29)	96	0.000	0.003
Asia	4	15,669	73,041	2.04 (1.58.2.63)	31.9	0.221	
America	1	937	10,839	1.07 (0.81.1.42)	0	-	
**NOS**							
**<**7	3	1,179,859	385,832	2.61 (1.48.4.60)	93.6	0	0.136
**≥**7	10	1,853,941	1.12231	1.59 (1.14.2.20)	95.5	0.000	
**Lag time**							
**<**1 year	2	4,570	67,056	1.66 (1.15.2.40)	53.2	0.144	0.897
**≥**1 year	10	3,029,112	1,330,592	1.71 (1.31.2.23)	95.3	0.000	
**Rear of publication**							
**<**2010	3	20,531	278,863	1.39 (1.19.1.61)	0	0.573	0.027
**≥**2010	10	3,013,269	119,200	1.94 (1.50.2.50)	94.4	0.000	
**Duration of PPI use**							
**<**1 year	4	N/A	N/A	1.56 (1.30.1.80)	0	0.486	0.493
1–3 years	3	N/A	N/A	1.75 (1.28.2.37)	0	0.421	
**>**3 years	4	N/A	N/A	2.32 (1.15, 4.66)	75.7	0.006	
**GC site**							
cardia	6	N/A	N/A	1.72 (1.19.2.48)	92.5	0.000	0.131
adenocarcinoma	2	N/A	N/A	2.04 (0.73.5.73)	95.4	0.000	
non-cardia	6	N/A	N/A	2.75 (2.09.3.62)	89	0.000	

### 3.4 Association of PPI use with colorectal cancer risk

The relationship between long-term PPI use and colorectal cancer risk is shown in Figure 6 in the Supplement appendix 3. The results of 11 studies conducted on 3,634,486 participants showed that long-term use of PPI had no significant correlation with the increased risk of colorectal cancer. The overall adjusted RR was 1.22 (95% CI: 0.95–1.55), which was highly heterogeneous (*p* < 0.05; I^2^ = 96.7%).

**FIGURE 6:**
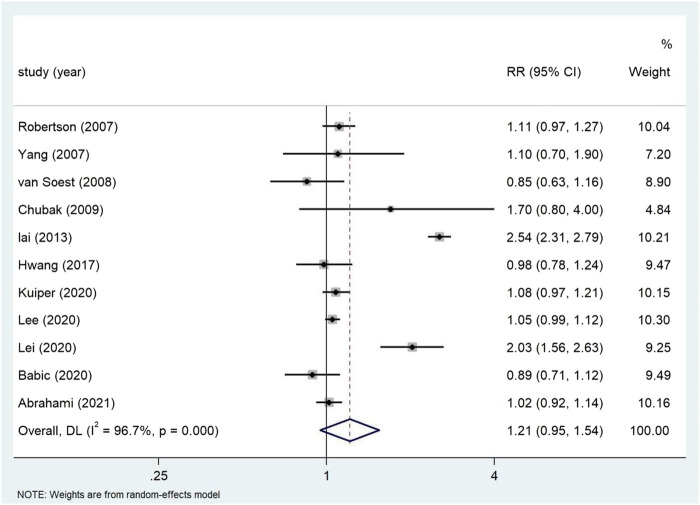
Forest plot of long-term use of PPI and the risk of Colorectal Cancer.

**FIGURE 7:**
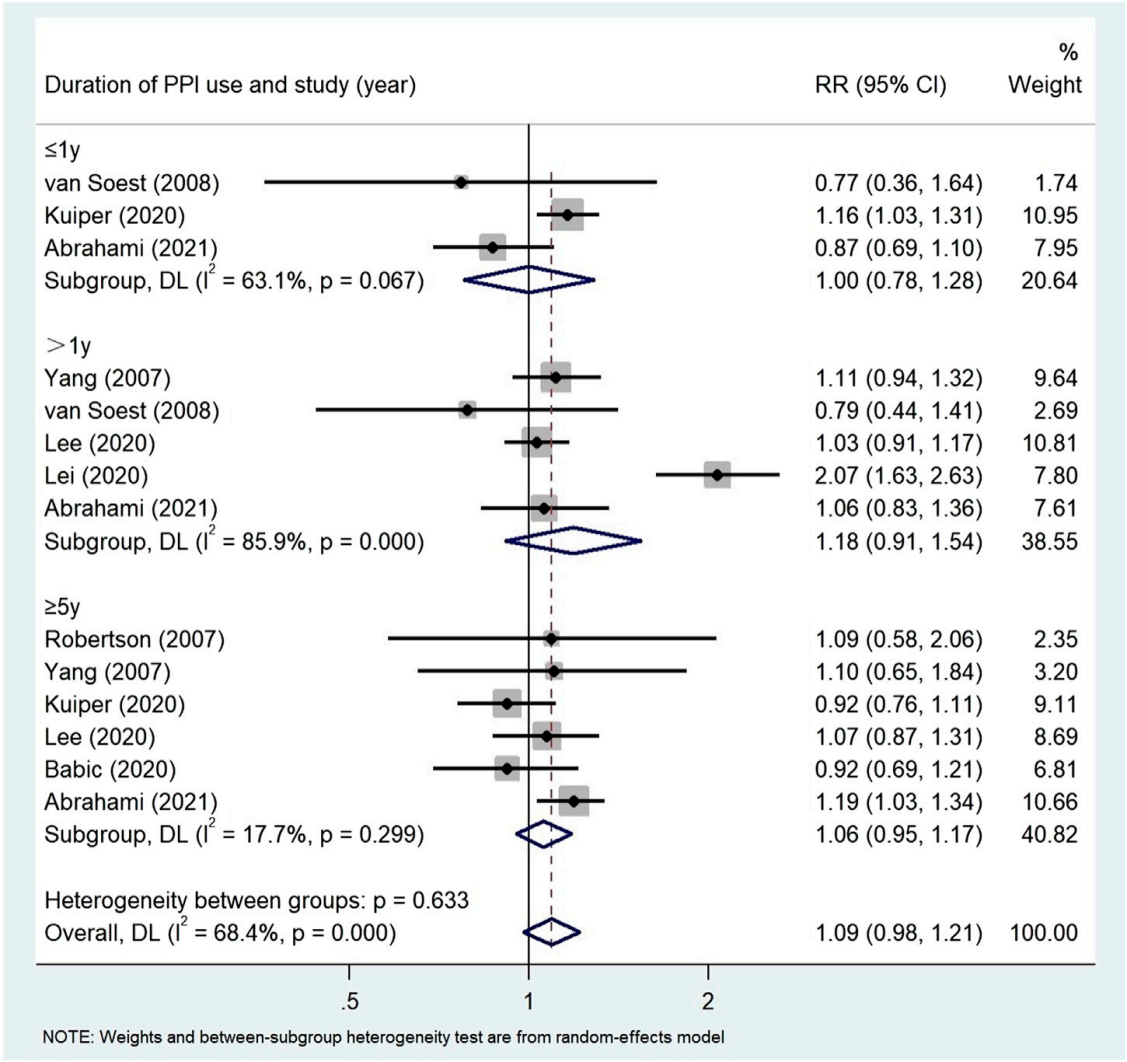
Association between the use of PPI and risks of Colorectal Cancer stratified by duration of PPI.

Subgroup analysis (Table 4) showed that there was no correlation between PPI use and CRC risk in more than 10,000 participants (RR = 1.74, 95% CI: 0.77–3.93). PPI use was significantly associated with colorectal cancer risk in Asian populations (RR = 1.72, 95% CI: 0.96–3.09). However, no evidence was found that PPI was associated with colorectal cancer risk in the United States (RR = 1.02, 95% CI: 0.87–1.19, *p* = 0.190) and Europe (RR = 1.06, 95% CI: 0.99–1.13). The duration of PPI was not significantly associated with the risk of colorectal cancer (≤1 year RR = 1.00, 95% CI: 0.78–1.28; >1 year RR = 1.18, 95% CI: 0.91–1.54; ≥5 years RR = 1.06, 95% CI: 0.95–1.17) (Figure 7). Among the 11 studies we included, only one nested case-control study (Lai et al., 2013) from Taiwan was of low quality and showed significant correlation (RR = 1.70, 95% CI: 0.95–1.55). In the subgroup analysis of high-quality studies, there was no significant correlation between the use of PPI and CRC risk (RR = 1.19, 95% CI: 0.93–1.54). Sensitivity analyses showed that the Lai (Lai et al., 2013) and Lee (Lee et al., 2020) studies had the greatest impact on the overall results (Supplementary Figure S1). After rejecting the literature for Lee and Lai, the RR was 1.11 (95% CI: 0.97–1.28), and the results still suggest that long-term use of PPI is associated with the risk of developing gastric cancer. However, the summary estimate still indicated not significantly associated with the risk of colorectal cancer. We found no evidence of publication bias in the Egger’s test and Begg’s test results (*p*_Egger’s test_ = 0.991, *p*_Begg’s test_ = 0.087).

**TABLE 4:** Subgroup analysis of Proton Pump Inhibitor use and the Risk of Colorectal Cancer.

Stratified by study design	NO. Of studies	Relative risk (95% CI)	I2 (%)	*p*-Value	Heterogenerty, *p*-value
case-control	3	1.07 (0.87.1.31)	14	0.312	0.695
Nested case-control	4	1.36 (0.81.2.28)	98.8	0.000	
Cohort	4	1.15 (0.85.1.54)	88.9	0.000	
**Events**					
**<**1,000	4	1.31 (0.85.2.04)	86.1	0.000	0.682
**≥**1,000	7	1.17 (0.85.1.62)	97.8	0.000	
**Population**					
**≥**10,000	2	1.74 (0.87.1.31)	90.4	0.001	0.534
**<**10,000	3	1.07 (0.77.3.93)	14.0	0.312	
**>**50,000	6	1.11 (0.97.1.27)	81.8	0.000	
**Mean age**					
**<**65	4	1.19 (0.84.1.68)	88.6	0.000	0.784
**≥**65	6	1.28 (0.87.1.87)	98.1	0.000	
**Region**					
Europe	5	1.06 (0.99.1.13)	0.0	0.726	0.233
Asia	3	1.72 (0.96.3.09)	96.4	0.000	
America	3	1.02 (0.87.1.19)	39.7	0.190	
**NOS**					
**<**7	1	1.70 (0.95.1.55)	0.0	-	0.412
**≥**7	10	1.19 (0.93.1.54)	97.0	0.000	
**Rear of publication**					
**<**2010	4	1.10 (0.97.1.25)	0.0	0.497	0.428
**≥**2010	7	1.27 (0.92.1.73)	98	0.000	
**Adjusted for CRC risk**					
Use of NSAIDs/aspirin	4	1.32 (0.80.2.18)	98.4	0.000	-
Body mass index	3	0.99 (0.91.1.09)	0.0	0.566	-
follow-up duration	2	1.10 (0.52.2.31)	56.3	0.13	-
**Duration of PPI use**					
**≤**1 year	3	1.00 (0.78.1.28)	63.1	0.067	0.633
**>**1 year	5	1.18 (0.91.1.54)	85.9	0	
**≥**5 years	6	1.06 (0.95, 1.17)	17.7	0.299	

## 4 Discussion

### 4.1 Proton pump inhibitor use and risk of gastric cancer

This meta-analysis included 12 studies on proton pump inhibitors and the risk of gastric cancer. We found that PPI was associated with 82% increased risk of gastric cancer. This is consistent with the research results of Zeng et al. (2021) (RR = 1.78, 95% CI: 1.38–2.31). Compared with the latest meta-analysis (Peng et al., 2022), we excluded database duplication and specific population studies, so our research results are more robust and accurate. The results of subgroup analysis showed that the risk of gastric cancer increased significantly with the extension of PPI prescription time, and the patients who used PPI for more than 3 years had the highest risk of gastric cancer. This result may be related to hypergastrinemia. Long term use of PPI leads to hypergastrinemia through strong acid inhibition. Long term hypergastrinemia may lead to gastric cancer associated with intestinal chromaffin cell proliferation (Delle Fave et al., 1998). It is well known that gastritis caused by *H. pylori* infection is an important factor leading to gastric cancer. Chronic *H. pylori* infection may induce gastric neuroendocrine tumours under the condition of hypergastrinemia (Rais et al., 2022). Subgroup analysis showed that the use of PPI and the risk of gastric cancer increased nearly threefold after eradication of potential confounders of *H. pylori* infection. Long term use of proton pump inhibitors after eradication of *H. pylori* may create a gastric environment for N-nitrosamine formation and gastric cancer development (Kobayashi et al., 2019). One well-known role of nitrosamines is to increase the risk of gastric adenocarcinoma (Corleto et al., 2014). According to the GC site stratification, the long-term PPI users had a high risk of non-cardia cancer, while the increased risk of cardiac cancer and adenocarcinoma was not obvious. Cardiac cancer is considered to be different from non-cardia cancer in etiology. Most non-cardiac gastric cancer is associated with atrophic gastritis and peptic ulcer caused by *helicobacter pylori* infection (Hansen et al., 2007). This may explain the correlation between the long-term use of PPI and the occurrence of non-cardiac cancer. Cardiac cancer may be related to gastroesophageal reflux and obesity. Distal esophageal adenocarcinoma and cardiac cancer share a common precancerous background of intestinal metaplasia caused by chronic inflammation, so it is difficult to distinguish them in epidemiological studies (Imamura et al., 2021). Gastroesophageal reflux disease is one of the indications of PPI, which may cause significant confusion. The real effect of PPI on the risk of cardiac cancer may be weakened by the treatment of gastroesophageal reflux, which is a common risk factor for distal esophageal adenocarcinoma. This may be the reason why the risk of cardiac cancer is not significantly increased.

### 4.2 Proton pump inhibitor use and risk of colorectal cancer

The results from 11 observational studies showed that use of PPI did not significantly increase the risk of colorectal cancer (RR = 1.22, 95% CI: 0.95, 1.55). This is consistent with Ma et al. (Ma et al., 2020) (RR = 1.26, 95% CI: 0.90–1.73). Compared with the previous meta-analysis, our study included three additional studies and more than two million additional patients. This is the most comprehensive study to investigate the relationship between PPI and risk of CRC. Seeing that the number of colorectal cancer participants from different regions and a wide range of subgroups, it is possible to conduct a more robust hierarchical analysis based on a standardized time frame that adjusted for CRC risk and PPI duration. The results of subgroup analysis showed that the risk of colorectal cancer did not increase significantly with the extension of PPI duration. This is the same as Ann et al. (Ahn et al., 2012). However, this is contrary to the findings of Abrahami et al. (Abrahami et al., 2022a). This may be due to the different characteristics of the study design and different study populations, as well as some uncontrollable confounding factors, so this result should be carefully interpreted. The nine studies included in this system review did not provide convincing evidence to prove the causal relationship between PPI use and CRC, and only two studies (Lai et al., 2013; Lei et al., 2021) found that there was a correlation between PPI and CRC. There is a research mechanism indicating that there is a biological relationship between the use of PPI and the risk of colorectal cancer. First of all, gastrin is an effective growth factor for normal and malignant gastrointestinal tissues, and has nutritional and tumorigenic effects. Secondly, long-term use of PPI will also lead to imbalance of intestinal microflora, decrease of abundance and diversity of intestinal microflora, and increase of pathogenic bacteria related to CRC carcinogenesis (Jackson et al., 2016; Si et al., 2021), which is conducive to the transformation and progress of colorectal cancer. However, there is also conflicting evidence that PPI may play an anti-tumor role through anti-inflammatory, antioxidant and antimutagenic mechanisms (Kim et al., 2010; Han et al., 2014).

It is worth noting that the early symptoms of gastric cancer are different from the late symptoms. Gastric cancer may have existed before PPI was used. Because of vague symptoms, including acid regurgitation, abdominal pain and heartburn, patients were treated with PPI before being diagnosed with gastric cancer. However, colorectal cancer does not cause reflux like symptoms. Therefore, patients with colorectal cancer usually do not need PPI treatment before being diagnosed with colorectal cancer. The significant association between the use of PPI and the increased risk of gastric cancer may indicate the contingency of clinical medication, rather than a causal relationship.

### 4.3 Study strengths and limitations

Our meta-analysis has several advantages. In this meta-analysis, the quality of the included studies was strictly evaluated, and the adjusted aggregate effect values were used to ensure the robustness of the results. Our study is the most comprehensive meta-analysis among the retrospective studies on the relationship between PPI use and the risk of gastric cancer and colorectal cancer compared with the previous systematic review and meta-analysis, including a large number of studies and participants.

There are some limitations in our study. There are still many potential uncontrollable confounding factors in our studies. The original study we included did not report the relationship between PPI dose and gastric cancer, and could not conduct subgroup analysis on specific the cumulative defined daily dose intervals. Biological gradient (dose response) is one of the important criteria for Bradford Hill to confirm causality. Our study does not meet the Bradford Hill criteria, so causality cannot be confirmed. It’ difficult to group and analysis based on the individual type of PPI, because most of the studies in these analyses do not report the type of PPI used. In addition, many of the studies we included did not provide information on over-the-counter PPI and precancerous lesions, which may affect the assessment.

## 5 Conclusion

Our meta-analysis showed that the use of PPI was associated with an 82% increased risk of gastric cancer, but we did not find that it was significantly associated with colorectal cancer. The duration dependent effect of PPI use has a significant positive correlation trend with the risk of gastric cancer and colorectal cancer, in which *H. pylori* infection may be a separate or synergistic risk. These results may be biased due to confounding factors such as PPI dose and type of PPI. Therefore, the potential relationship between PPI related gastric cancer and colorectal cancer needs further validation and support from more prospective studies. The indication of PPI treatment should be verified, and the lowest effective dose should be used for PPI in the shortest possible time or the use of PPI should be avoided.

## Data Availability

The original contributions presented in the study are included in the article/Supplementary Material, further inquiries can be directed to the corresponding author.
