# Supplementary material for: Association of proton pump inhibitors with gastric and colorectal cancer risk: A systematic review and meta-analysis
Source: Front Pharmacol. 2023 Mar 16;14:1129948. doi: 10.3389/fphar.2023.1129948 (PMC10060974; doi:10.3389/fphar.2023.1129948)

Supplementary Material

Association of proton pump inhibitors with gastric and colorectal cancer risk: a systematic review and meta-analysis

# Supplementary File S1. Literature search strategy for the databases

1.Search strategy for PubMed from inception to March 21, 2022

(Stomach Neoplasms OR Neoplasms, Stomach OR Gastric Neoplasms OR Neoplasms, Gastric OR Stomach Cancers OR Gastric Cancers OR gastric adencarcinoma OR Cardiac cancer OR stomach tumor) AND (Proton Pump Inhibitors OR ppis OR Omeprazole OR pantoprazole OR Lansoprazole OR Esomeprazole OR Rabeprazole)

(colorectal cancer OR colon cancer OR rectal cancer OR colorectal adenocarcinoma OR colon adenocarcinoma OR rectal adenocarcinoma) AND (proton pump inhibitor OR PPI OR pantoprazole OR omeprazole OR esomeprazole OR lansoprazole OR ilaprazole)

2.Search strategy for Embase from inception to March 21, 2022

('stomach neoplasms':ti,ab,kw OR 'stomach tumor acid':ti,ab,kw OR 'stomach cancer ':ti,ab,kw OR 'gastric tumor':ti,ab,kw OR 'gastric neoplasm':ti,ab,kw OR 'gastric cancer':ti,ab,kw) AND ('proton pump inhibitor':ti,ab,kw OR 'PPI':ti,ab,kw OR 'pantoprazole':ti,ab,kw OR 'omeprazole':ti,ab,kw OR 'esomeprazole':ti,ab,kw OR 'lansoprazole':ti,ab,kw OR 'ilaprazole':ti,ab,kw)

('colorectal cancer':ti,ab,kw OR 'colon cancer':ti,ab,kw OR 'rectal cancer':ti,ab,kw OR 'colorectal adenocarcinoma':ti,ab,kw OR 'rectal adenocarcinoma':ti,ab,kw) AND ('proton pump inhibitor':ti,ab,kw OR 'PPI':ti,ab,kw OR 'pantoprazole':ti,ab,kw OR 'omeprazole':ti,ab,kw OR 'esomeprazole':ti,ab,kw OR 'lansoprazole':ti,ab,kw OR 'ilaprazole':ti,ab,kw)

3.Search strategy for Web of Science from inception to March 21, 2022

#1 TS= (Stomach Neoplasms OR Neoplasms, Stomach OR Gastric Neoplasms OR Neoplasms, Gastric OR Stomach Cancers OR Gastric Cancers OR gastric adencarcinoma OR Cardiac cancer OR stomach tumor)

#2 TS= (colorectal cancer OR colon cancer OR rectal cancer OR colorectal adenocarcinoma OR colon adenocarcinoma OR rectal adenocarcinoma)

#3 TS= (Proton Pump Inhibitors OR ppis OR Omeprazole OR pantoprazole OR Lansoprazole OR Esomeprazole OR Rabeprazole)

#1 #2 AND #3

4. Search strategy for Cochrane library from inception to March 21, 2022

(stomach neoplasms OR stomach tumor OR stomach neoplasm OR stomach cancer OR gastric tumor OR gastric neoplasm OR gastric cancer) AND (proton pump inhibitor OR PPI)

(colorectal cancer OR colon cancer OR rectal cancer OR colorectal adenocarcinoma OR colon adenocarcinoma OR rectal adenocarcinoma) AND (proton pump inhibitor OR PPI OR pantoprazole OR omeprazole OR esomeprazole OR lansoprazole OR ilaprazole)

###### Supplementary table S1. Newcastle-Ottawa scale scores and quality assessment: (a)case-control studies (b) cohort studies

**(a)**

| **Study  (first author, year)** | **Selection** | | | | **Comparability** | | **Exposure** | | | |
| --- | --- | --- | --- | --- | --- | --- | --- | --- | --- | --- |
|  | **Adequacy** | **Represen- tiveness** | **Selection** | **Definition** | **Main  factors** | **Additional  factors** | **Ascertain- ment** | **Method** | **Non-response** | **Quality** |
| Garcia,2006 | ★ | ★ | ★ | ★ | ★ | ★ | ★ | ★ | ☆ | 8 |
| Yang,2007 | ☆ | ★ | ★ | ★ | ★ | ★ | ★ | ★ | ☆ | 7 |
| Robertson,2007 | ☆ | ★ | ★ | ★ | ★ | ★ | ★ | ★ | ☆ | 7 |
| van,2008 | ★ | ★ | ★ | ★ | ★ | ★ | ★ | ★ | ☆ | 8 |
| Tamim, 2008 | ☆ | ★ | ★ | ★ | ★ | ★ | ★ | ★ | ☆ | 7 |
| Chubak,2009 | ☆ | ★ | ★ | ★ | ★ | ☆ | ★ | ★ | ☆ | 6 |
| Lai, 2013 | ☆ | ★ | ★ | ★ | ★ | ★ | ★ | ★ | ☆ | 7 |
| Lai, 2018 | ☆ | ★ | ★ | ★ | ★ | ☆ | ★ | ★ | ☆ | 6 |
| Liu(PCCIU),2020 | ☆ | ★ | ★ | ★ | ★ | ★ | ★ | ★ | ☆ | 7 |
| kuiper,2020 | ☆ | ★ | ★ | ★ | ★ | ★ | ★ | ★ | ☆ | 7 |
| Lee, 2020 | ☆ | ★ | ★ | ★ | ★ | ★ | ★ | ★ | ☆ | 7 |

**(b)**

| **Study  (first author,  year)** | **Selection** | | | | **Comparability** | | **outcome** | | | |
| --- | --- | --- | --- | --- | --- | --- | --- | --- | --- | --- |
|  | **Represen- tiveness** | **Selection** | **Ascertain- ment** | **Outcome** | **Main  factor** | **Additional  factor** | **Assessment** | **Follow-up** | **Adequacy** | **Quality** |
| Poulsen,2009 | ★ | ★ | ★ | ★ | ★ | ★ | ★ | ☆ | ★ | 8 |
| Cheung,2017 | ★ | ☆ | ★ | ★ | ★ | ★ | ★ | ★ | ★ | 8 |
| Wennerström,2017 | ★ | ★ | ★ | ☆ | ★ | ☆ | ★ | ★ | ★ | 7 |
| Niikura,2017 | ☆ | ★ | ☆ | ☆ | ★ | ★ | ☆ | ★ | ★ | 5 |
| Hwang,2017 | ★ | ★ | ★ | ★ | ★ | ★ | ★ | ★ | ★ | 9 |
| Brusselaers,2019 | ★ | ★ | ★ | ★ | ★ | ☆ | ★ | ★ | ★ | 8 |
| Liu (UK),2020 | ★ | ★ | ★ | ★ | ★ | ★ | ★ | ☆ | ☆ | 7 |
| Lei, 2020 | ★ | ★ | ★ | ★ | ★ | ★ | ★ | ☆ | ☆ | 7 |
| Babic,2020 | ★ | ★ | ☆ | ★ | ★ | ★ | ★ | ★ | ★ | 8 |
| Seo,2021 | ★ | ★ | ★ | ★ | ★ | ★ | ★ | ★ | ☆ | 8 |
| Abrahami',2021 | ★ | ☆ | ★ | ★ | ★ | ★ | ★ | ★ | ★ | 8 |
| Abrahami,2021 | ★ | ☆ | ★ | ★ | ★ | ★ | ★ | ★ | ★ | 8 |

###### Supplementary figure S1. Sensitivity analysis of included studies:(a) gastric cancer (b)colorectal cancer

**(a)**

**
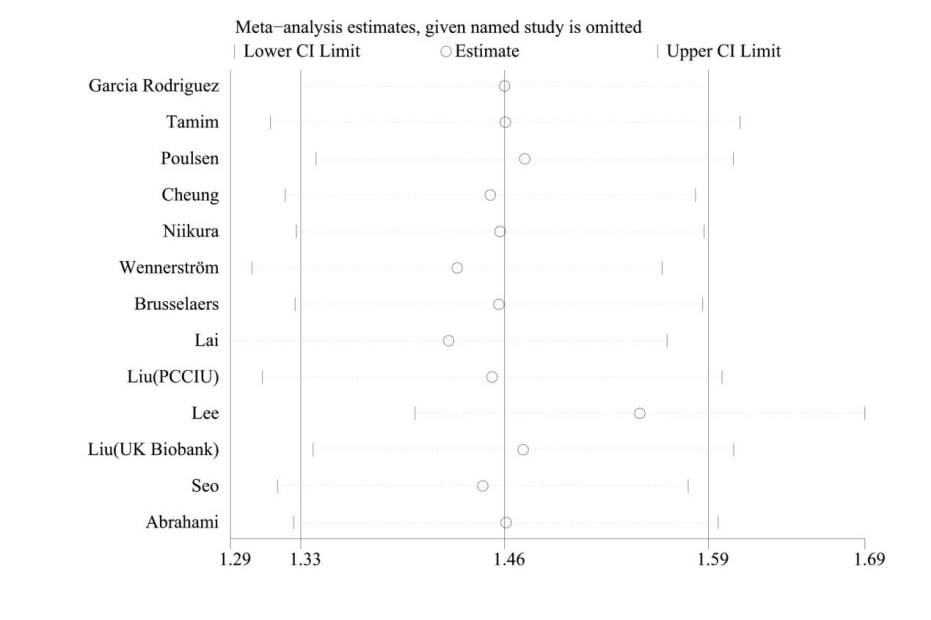
**

**(b)**


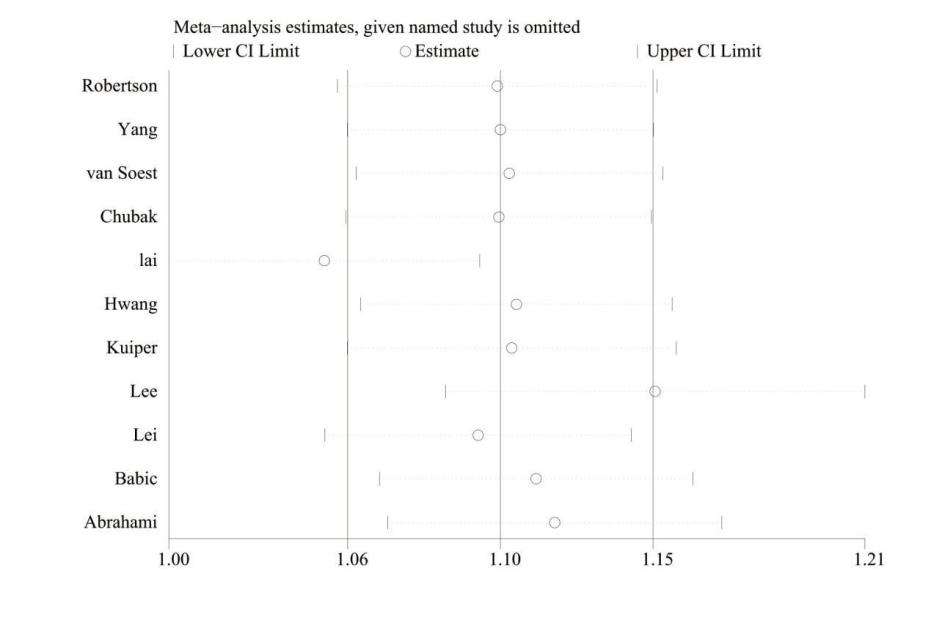

Supplement: Supplementary file 1 [file DataSheet1.docx]
